# The Association of *ADORA2A* and *ADORA2B* Polymorphisms with the Risk and Severity of Chronic Heart Failure: A Case-Control Study of a Northern Chinese Population

**DOI:** 10.3390/ijms16022732

**Published:** 2015-01-26

**Authors:** Ya-Jing Zhai, Ping Liu, Hai-Rong He, Xiao-Wei Zheng, Yan Wang, Qian-Ting Yang, Ya-Lin Dong, Jun Lu

**Affiliations:** 1 Department of Pharmacy, the First Affiliated Hospital of Medical College, Xi’an Jiaotong University, Xi’an 710061, China; E-Mails: zhaiyajing1991@163.com (Y.-J.Z.); hehairong19891989@126.com (H.-R.H.); zhengxiaowei_1990@163.com (X.-W.Z.); wangyan910819@gmail.com (Y.W.); yangqiangting12@126.com (Q.-T.Y.); 2 Department of Cardiovascular Medicine, the First Affiliated Hospital of Medical College, Xi’an Jiaotong University, Xi’an 710061, China; E-Mail: pingdoctor@163.com

**Keywords:** *ADORA2A*, *ADORA2B*, chronic heart failure, single nucleotide polymorphism, rs4822489

## Abstract

The causes of chronic heart failure (CHF) and its progression are likely to be due to complex genetic factors. Adenosine receptors A2A and A2B (ADORA2A and ADORA2B, respectively) play an important role in cardio-protection. Therefore, polymorphisms in the genes encoding those receptors may affect the risk and severity of CHF. This study was a case-control comparative investigation of 300 northern Chinese Han CHF patients and 400 ethnicity-matched healthy controls. Four common single-nucleotide polymorphisms (SNPs) of *ADORA2A* (rs2236625, rs2236624, rs4822489, and rs5751876) and one SNP of *ADORA2B* (rs7208480) were genotyped and an association between SNPs and clinical outcomes was evaluated. Odds ratios (ORs) with 95% confidence intervals (CIs) were used to assess the association. The rs4822489 was significantly associated with the severity of CHF after adjustment for traditional cardiovascular risk factors (*p* = 0.040, OR = 1.912, 95% CI = 1.029–3.550). However, the five SNPs as well as the haplotypes were not found to be associated with CHF susceptibility. The findings of this study suggest that rs4822489 may contribute to the severity of CHF in the northern Chinese. However, further studies performed in larger populations and aimed at better defining the role of this gene are required.

## 1. Introduction

Chronic heart failure (CHF) is the end result of various insults to the myocardium, the most common of which is ischemic heart disease [1]. The prevalence of CHF is high in older individuals, and it is a major cause of morbidity, mortality, hospitalizations, and disability [2]. Typically 1%–2% of the adult population in developed countries suffers from CHF, with the prevalence rising to ≥10% among persons aged ≥70 years [3]. The pathogenesis of CHF and genetic factors underlying the predisposition to it remain largely unknown. Identification of the genes associated with CHF is important to improve the understanding of the etiology of this multifactorial disorder.

Adenosine is a naturally occurring endogenous purine nucleoside that is composed of an adenine molecule attached to a ribose sugar moiety (ribofuranose) via a β-N9-glycosidic bond (6-amino-9-β-d-ribofuranosyl-9-H-purine) [4]. Levels of adenosine increase in response to physiological and pathological stimuli. The resultant shifts in adenosine levels impact all major aspects of cardiovascular function, including the rate and strength of the heartbeat, conduction of the cardiac impulse, autonomic control of the heart, coronary perfusion, cardiovascular growth or remodeling, and cardiac and vascular resistance to injurious insult [5].

Adenosine regulates cell function via four structurally related G-protein-coupled receptors: A_1_ (ADORA1), A_2_ (ADORA2), and A_3_ (ADORA3). In addition, ADORA2 was subsequently sub-divided according to the existence of distinct high-(ADORA2A)* vs.* low-affinity (ADORA2B) binding sites. Layland* et al.* [6] revealed that the use of adenosine for stress testing and induction of systemic (and coronary) hyperemia is primarily related to the activation of *ADORA2A* and the resultant increase in myocardial blood flow. Furthermore, deletion of the *ADORA2A* gene has been shown to reduce the heart rate [7], and in a head-to-head comparison in gene-targeted mice for all four individual adenosine receptors, researchers observed that mice deficient in the gene encoding *ADORA2B* are particularly prone to myocardial ischemia [8,9,10,11,12,13]. Overall, *ADORA2A* and *ADORA2B* play an important role in heart activity.

The activities of *ADORA2A* and *ADORA2B* could be affected by gene polymorphisms* in vivo*. It is therefore reasonable to hypothesize that single-nucleotide polymorphisms (SNPs) in ADORA2A and ADORA2B can influence the activity of the adenosine A_2_ receptors, and further affect the susceptibility and progression of CHF. To the best of our knowledge, this hypothesis has not been investigated previously. We therefore investigated whether four selected *ADORA2A* SNPs (rs2236625, rs2236624, rs4822489, and rs5751876) and one *ADORA2B* SNP (rs7208480) are associated with CHF risk or severity in a north Chinese population.

## 2. Results

### 2.1. Basic Characteristics

The baseline characteristics of the study subjects are listed in Table 1. The age and gender distributions did not differ significantly between the healthy controls and the CHF patients. The prevalence of all traditional cardiovascular risk factors was significantly higher among the CHF patients than in the healthy control group (*p* < 0.05).

**Table 1:** Demographic and clinical characteristics of study populations.

Factors	CHF (*n* = 300)	Controls (*n* = 400)	*p* Value
Age (years)	61.41 ± 12.506	60.33 ± 8.558	0.177 ^a^
Males/females	175/125	211/189	0.142 ^b^
Hypertension	172 (57.33%)	122 (30.50%)	<0.001 ^b^
Dyslipidemia	84 (28.00%)	81 (20.25%)	0.017 ^b^
Diabetes	104 (34.67%)	67 (16.75%)	<0.001 ^b^
Smoking	91 (30.33%)	62 (15.50%)	<0.001 ^b^
NYHA class			
II	94 (31.33%)		
III	117 (39.00%)		
IV	89 (29.67%)		

CHF, chronic heart failure; NYHA, New York Heart Association. ^a^ *p* values were calculated by separate variance estimation *t*-test as variance between the two group is not neat; ^b^ *p* value was calculated from two-sided χ^2^-test.

### 2.2. Association of ADORA2A and ADORA2B Genotypes with the Susceptibility of CHF

The results of the genotype distribution and allele frequencies of the five SNPs for the entire case-control group are given in Table 2. The genotype distribution for all SNPs except rs7208480 did not deviate from HWE in the control group (*p* > 0.05). The allele frequencies and genotype distribution of these five SNPs did not differ significantly between the CHF patients and healthy controls in a univariate analysis. After adjustment for age, sex, and traditional cardiovascular risk factors, there was still no evidence that the risk of CHF either increased or decreased significantly in the presence of any of the SNPs (Table 3).

Linkage disequilibrium (LD) analysis for the *ADORA2A* gene showed that three SNPs (rs4822489, rs2236624, rs2236625) were in strong LD with each other (*D*' > 0.87, *R*^2^ > 0.03). Then, the association between inferred haplotypes and CHF risk among the individuals was analyzed. But no haplotype was found to be associated with the CHF susceptibility (Table 4).

The genotype distribution and allele frequencies of the five SNPs were investigated in relation to a CHF clinical subset (Table 5). A significantly higher prevalence of the rs2236625 CC genotype and C allele was observed in smokers among CHF patients (*p* = 0.047 and 0.025, respectively).

**Table 2:** Genotype distribution of the SNPs and their associations with the risk of CHF under different contrast models.

Genotype	Controls *n* (%)	CHF *n* (%)	HWE *p* ^a^	Dominant Model *p* ^a^	Recessive Model *p* ^a^	Additive Model *p* ^a^	Allele Contrast *p* ^a^
rs2236624			0.752	0.842	0.223	0.390	0.381
TT (W)	33 (8.27)	26 (8.70)					
CT	159 (39.85)	104 (34.78)					
CC	207 (51.88)	169 (56.52)					
rs2236625			0.319	0.699	0.443	0.433	0.932
CC (W)	312 (78.39)	238 (79.60)					
CT	83 (20.85)	56 (18.73)					
TT	3 (0.75)	5 (1.67)					
rs4822489			0.219	0.450	0.729	0.609	0.814
TT (W)	98 (24.69)	66 (22.22)					
GT	186 (46.85)	150 (50.51)					
GG	113 (28.46)	81 (27.27)					
rs5751876			0.996	0.818	0.445	0.650	0.725
TT (W)	90 (22.67)	70 (23.41)					
CT	198 (49.87)	139 (46.49)					
CC	109 (27.46)	90 (30.10)					
rs7208480			<0.001	0.283	0.555	0.760	0.322
CC (W)	197 (49.25)	135 (45.15)					
CT	195 (48.75)	156 (52.17)					
TT	8 (2.00)	8 (2.68)					

Abbreviations: CHF, chronic heart failure; HWE, Hardy–Weinberg equilibrium. ^a^* p* values were calculated from a two-sided *χ*^2^-test.

**Table 3:** Logistic regression analysis for SNPs under different genetic models, adjusted for age, sex, and cardiovascular risk factors.

SNPs	Dominant Model	Recessive Model	Additive Model
rs2236624			
Odds ratio (95% CI)	0.815 (0.458–1.449)	1.243 (0.900–1.717)	1.097 (0.854–1.410)
*p*	0.485	0.186	0.468
rs2236625			
Odds ratio (95% CI)	1.072 (0.724–1.588)	1.856 (0.419–8.231)	1.101 (0.768–1.578)
*p*	0.729	0.416	0.601
rs4822489			
Odds ratio (95% CI)	1.062 (0.725–1.554)	0.953 (0.664–1.368)	1.002 (0.767–1.205)
*p*	0.757	0.794	0.984
rs5751876			
Odds ratio (95% CI)	0.790 (0.538–1.159)	1.111 (0.778–1.586)	0.961 (0.767–1.205)
*p*	0.228	0.563	0.732
rs7208480			
Odds ratio (95% CI)	1.245 (0.902–1.718)	1.720 (0.599–4.942)	1.257 (0.934–1.691)
*p*	0.182	0.314	0.131

**Table 4:** Associations between haplotypes of three* ADORA2A* variants (rs4822489, rs2236624, rs2236625) and the risk of CHF.

Haplotypes ^a^	CHF *n* (%)	Control *n* (%)	Without Adjustment		With Adjustment	
			*p* ^b^	OR (95% CI)	*p* ^c^	OR (95% CI)
G-C-C	312.5 (52.06)	412.0 (51.89)	-	1.00	-	1.00
T-T-C	153.0 (25.76)	225.0 (28.34)	0.42	1.11 (0.87–1.42)	0.53	1.09 (0.84–1.42)
T-C-T	63.0 (10.61)	89.0 (11.21)	0.72	1.07 (0.75–1.53)	0.80	0.95 (0.65–1.39)
T-C-C	65.5 (11.03)	68.0 (8.56)	0.20	0.78 (0.54–1.14)	0.33	0.82 (0.54–1.22)

Bonferroni’s multiple adjustment was applied to the level of significance, which was set at *p* < 0.0125 (0.05/4). ^a^ SNPs of haplotype are (in sequence) rs4822489, rs2236624, and rs2236625. Frequency less than 0.05 will be ignored in analysis; ^b^ *p* values were calculated from two-sided chi-square tests or Fisher’s exact tests; ^c^ *p* values were calculated by unconditional logistic regression, adjusted for age, sex, and cardiovascular risk factors.

**Table 5:** SNP genotype distribution and allele frequency, according to subsets, in CHF patients.

Genotype	Hypertension *n* = 172 (%)	Dyslipidemia *n* = 84 (%)	Diabetes *n* = 104 (%)	Smoking Habit *n* = 90 (%)
rs2236624				
TT	14 (8.14)	9 (10.71)	12 (11.54)	9 (10.00)
CT	60 (34.88)	34 (40.48)	38 (35.54)	33 (36.67)
CC	98 (56.98)	41 (48.81)	54 (51.92)	48 (53.33)
Allele frequency *p*-value	0.743 ^a^	0.090 ^a^	0.130 ^a^	0.412 ^a^
Genotype distribution* p*-value	0.923 ^a^	0.239 ^a^	0.330 ^a^	0.735 ^a^
rs2236625				
CC	139 (80.8)	67 (79.76)	86 (82.69)	78 (86.67)
CT	28 (16.28)	16 (19.05)	17 (16.35)	12 (13.33)
TT	5 (2.91)	1 (1.19)	1 (0.96)	0 (0.00)
Allele frequency *p*-value	0.993 ^a^	0.875 ^a^	0.278 ^a^	0.025 ^a^
Genotype distribution* p*-value	0.544 ^b^	0.965 ^b^	0.332 ^b^	0.047 ^b^
rs4822489				
TT	36 (21.18)	19 (22.62)	25 (24.27)	16 (18.18)
GT	83 (48.82)	48 (57.14)	50 (48.54)	53 (60.23)
GG	51 (30.00)	17 (20.24)	28 (27.18)	19 (21.59)
Allele frequency *p*-value	0.287 ^a^	0.255 ^a^	0.704 ^a^	0.795 ^a^
Genotype distribution* p*-value	0.472 ^a^	0.206 ^a^	0.811 ^a^	0.094 ^a^
rs5751876				
TT	40 (23.26)	16 (19.05)	18 (17.31)	17 (18.89)
CT	84 (48.84)	43 (51.19)	53 (50.96)	46 (51.11)
CC	48 (27.91)	25 (29.76)	33 (31.73)	27 (30.00)
Allele frequency *p*-value	0.561 ^a^	0.537 ^a^	0.166 ^a^	0.477 ^a^
Genotype distribution* p*-value	0.568 ^a^	0.470 ^a^	0.186 ^a^	0.424 ^a^
rs7208480				
CC	77 (44.77)	39 (46.43)	50 (48.08)	44 (48.89)
CT	91 (52.91)	43 (51.19)	53 (50.96)	44 (48.89)
TT	4 (2.32)	2 (2.38)	1 (0.96)	2 (2.22)
Allele frequency *p*-value	0.992 ^a^	0.791 ^a^	0.360 ^a^	0.457 ^a^
Genotype distribution* p*-value	0.887 ^a^	0.950 ^a^	0.350 ^a^	0.683 ^a^

^a^ χ^2^-test: allele frequency and genotype distribution among the three groups; ^b^ χ^2^-test: genotype distribution CC* vs.* CT + TT.

### 2.3. Association of ADORA2A and ADORA2B Genotypes with the Severity of CHF

Analysis of the association between the different genotype groups of each polymorphism and NYHA class in CHF patients revealed that the genotype distribution and allele frequencies differed significantly between rs4822489 and the NYHA class of the CHF patients (*p* = 0.024 and 0.020, respectively; Table 6). After univariate analysis, the *p* values of two variables (age and dyslipidemia) were <0.1 (*p* = 0.012 and 0.094, respectively), and thus they were entered into a multivariable ordinal regression model. After that, only rs4822489 was significantly associated with the severity of CHF (*p* = 0.040, odds ratio = 1.912, 95% CI = 1.029–3.550, GG* vs.* TT).

**Table 6:** SNP genotype distribution and allele frequency, according to NYHA class, in CHF patients.

Genotype	NYHA II	NYHA III	NYHA IV
rs2236624			
TT	6 (2.01%)	13 (4.35%)	7 (2.34%)
CT	41 (13.71%)	38 (12.71%)	25 (8.36%)
CC	46 (15.38%)	66 (22.07%)	57 (19.06%)
Allele frequency *p* value	0.307 ^a^		
Genotype distribution* p* value	0.150 ^a^		
rs2236625			
CC	67 (22.41%)	98 (32.78%)	73 (24.41%)
CT	25 (8.36%)	15 (5.02%)	16 (5.35%)
TT	1 (0.33%)	4 (1.34%)	0
Allele frequency* p* value	0.183 ^a^		
Genotype distribution* p* value	0.089 ^b^		
rs4822489			
TT	23 (7.74%)	27 (9.09%)	16 (5.39%)
GT	51 (17.17%)	62 (20.88%)	37 (12.46%)
GG	19 (6.40%)	26 (8.75%)	36 (12.12%)
Allele frequency *p* value	0.020 ^a^		
Genotype distribution* p* value	0.024 ^a^		
rs5751876			
TT	24 (8.03%)	29 (9.70%)	17 (5.69%)
CT	39 (13.04%)	56 (18.73%)	44 (14.72%)
CC	30 (10.03%)	32 (10.70%)	28 (9.36%)
Allele frequency *p* value	0.614 ^a^		
Genotype distribution *p* value	0.708 ^a^		
rs7208480			
CC	44 (14.72%)	53 (17.73%)	38 (12.71%)
CT	46 (15.38%)	63 (21.07%)	47 (15.72%)
TT	3 (1.00%)	1 (0.33%)	4 (1.34%)
Allele frequency* p* value	0.753 ^a^		
Genotype distribution *p* value	0.546 ^a^		

Abbreviations: NYHA, New York Heart Association. ^a^ χ^2^-test: allele frequency and genotype distribution among the three groups; ^b^ χ^2^-test: genotype distribution CC* vs.* CT + TT.

The group distributions of *ADORA2A* and *ADORA2B* genotypes were analyzed relative to the clinicopathological parameters related to CHF severity. None of these parameters differed significantly among the different genotype groups (Table 7).

**Table 7:** Parameters related to the severity of CHF in different genotype groups.

Genotype	EF Mean Value	LVEDD Mean Value	LVESD Mean Value
rs2236624			
TT	40.73 ± 14.70	64.40 ± 11.11	51.60 ± 14.01
CT	42.21 ± 13.10	64.64 ± 11.07	51.74 ± 12.79
CC	39.91 ± 112.51	65.18 ± 11.11	52.51 ± 12.54
*p* ^a^	0.359	0.902	0.871
rs2236625			
CC	40.68 ± 13.14	65.15 ± 11.12	52.34 ± 12.79
CT	40.93 ± 12.54	64.51 ± 11.15	51.95 ± 12.86
TT	44.00 ± 4.36	59.00 ± 5.96	46.20 ± 6.65
*p* ^a^	0.847	0.449	0.561
rs4822489			
TT	42.65 ± 14.47	63.51 ± 11.05	50.52 ± 13.64
GT	40.51 ± 12.01	64.68 ± 10.85	52.25 ± 12.20
GG	39.94 ± 13.27	66.21 ± 11.26	53.01 ± 12.75
*p* ^a^	0.411	0.339	0.492
rs5751876			
TT	42.54 ± 13.57	64.00 ± 11.13	51.12 ± 13.27
CT	40.00 ± 11.75	65.28 ± 11.08	52.76 ± 12.38
CC	42.54 ± 13.59	65.11 ± 11.06	52.07 ± 12.88
*p* ^a^	0.403	0.726	0.682
rs7208480			
CC	40.31 ± 12.04	65.18 ± 11.81	52.74 ± 12.96
CT	41.02 ± 13.14	64.85 ± 10.39	51.89 ± 12.40
TT	44.00 ± 22.14	62.13 ± 11.72	47.75 ± 15.30
*p* ^a^	0.696	0.747	0.523

Abbreviations: EF, ejection fraction; LVEDD, left-ventricle end-diastolic diameter; LVESD, left-ventricle end-systolic diameter. ^a^ *p* values were calculated from a two-sided Analysis of Variance; Multiple comparisons among pairs of means were also tested using Dunnett’s test and no significance was found.

## 3. Discussion

This is the first report on the impact of *ADORA2A* and *ADORA2B* polymorphisms on CHF susceptibility and progression in Chinese patients. After adjustment for age, sex, and traditional cardiovascular risk factors, no significant differences were found between CHF patients and controls. In addition, the possible haplotype associated with CHF risk was also not found by the haplotype analysis of the *ADORA2A* gene. However, the rs4822489 SNP in *ADORA2A* was significantly associated with NYHA class in CHF patients; this SNP may therefore affect the progression of CHF.

Adenosine is a potent coronary vasodilator that is produced in cardiac myocytes via the catabolism of adenine nucleotides [14]. It binds with four evolutionarily well-conserved receptors that are expressed ubiquitously [15,16]. Activation of *ADORA2A* and *ADORA2B* produces potent vasodilation of most vascular beds including the coronary circulation, resulting in an increase in myocardial blood flow [17]. Furthermore, *ADORA2A* indirectly alters cardiac contractility by modulating the ADORA1 antiadrenergic effect [18]. The gene encoding *ADORA2A* is located on chromosome 22q11.23. The rs4822489 SNP, for which the minor allele frequency is 0.467 in the Chinese Han population, is located in the intron areas of *ADORA2A*, thus causing no change of encoding protein in theory. However, an intronic SNP might alter the splicing of primary transcripts or gene expression [19]. Howe* et al.* [20] revealed that one specific subset that exerted distinct effects on a multifunctional intron retention reporter was likely preferred for the regulation of endogenous intron retention events. That is, intron retention could affect alternative splicing of pre-mRNA by influencing the exonic splicing silencers. Through alternative splicing, a gene could produce different mature mRNAs and then translated proteins with different functions, and in extreme cases with opposite functions [20,21,22]. Therefore, we hypothesized that the intronic SNP rs4822489 could affect the expression of *ADORA2A* by influencing the alternative splicing of its mRNA, leading to alteration of the number and function of *ADORA2A*, which might in turn weaken or eliminate the cardioprotective effects of that receptor, including expanding the coronary artery and increasing cardiac contractility and output, to ultimately aggravate any heart damage and accelerate the progression of heart failure. Another possible explanation of the association between the intronic rs4822489 and NYHA class could be that this SNP is in linkage disequilibrium with the “causal” genetic variant. 

It has been shown that the rs4822489 variant is associated with other diseases. Bashira* et al.* [23] explored the association between variants of *ADORA2A* and proliferative diabetic retinopathy (PDR) in a cohort of patients with type 1 diabetes. They found that carriers homozygous for the T allele of SNP rs4822489 were less likely to progress to PDR during follow-up than were carriers of any G allele. Hider* et al.* [24] examined the role of *ADORA2A* polymorphisms on the outcome of methotrexate (MTX) treatment in rheumatoid arthritis (RA), but they did not identify any association between rs4822489 and adverse events in RA patients for this SNP because of the genotyping success rate of <85%. Three other *ADORA2A* SNPs were detected in the present study: rs2236624, rs2236625, and rs5751876. rs2236624 is an intronic SNP and is in linkage disequilibrium with 15 currently known SNPs, 4 of which are within exons but are synonymous coding SNPs; 5 are intronic, 6 lie in the 3' untranslated region, 1 is a nonsynonymous SNP, and another is a frame-shift polymorphism [25]. Christine* et al.* [26] found that in both healthy people and patients with autism spectrum disorder, those with the rs2236624 CC genotype were more susceptible to anxiety and autism. Bashira* et al.* [23] concluded that type 1 diabetes patients with the rs2236624TT genotype were not susceptible to PDR. Hider* et al.* [24] considered that the rs2236624 T-allele was significantly associated with the adverse events of MTX treatment in RA. The rs5751876 SNP is a synonymous substitution located in a tyrosine codon of exon 2, which is reportedly associated with neuropsychiatric phenotypes [27,28,29] and age at onset in patients with Huntington’s disease [30,31]. In addition, Xiaoyan* et al.* [32] found that the frequency of heterozygotes with the rs5751876 genotype was significantly lower in subjects with a high degree of myopia. The rs5751876 SNP is also reportedly associated with anxiety [29,33,34]. However, there have been no relevant research reports for rs2236625.

*ADORA2B* is located on chromosome 21p12; the rs7208480 (intergenic) SNP of this gene was selected in this study to explore its effects on CHF. Desai* et al.* [35] concluded that rs7208480 was significantly associated with pulmonary hypertension (PH) and sickle cell disease. PH is common in patients with CHF and is associated with more severe symptoms and worse outcomes [36]. It is also a strong and independent predictor of mortality among patients with heart failure and provides incremental and clinically relevant prognostic information independently of known predictors of outcomes [37,38,39]. However, the present study found no association between the rs7208480 polymorphism and susceptibility to CHF and progression thereof.

In this study, we also found that the prevalence of the rs2236625 CC genotype and C allele were significantly higher in smokers among CHF patients, which suggested that this SNP might have a certain effect on the cardiovascular function of smokers. It is reported that adenosine could increase myocardial contractility, which might be due to increased coronary blood flow through activation of ADORA2A receptors (Gregg’s phenomenon) [40,41]. Smoking is known to be associated with the impairment of coronary endothelial function [42,43,44]. Therefore, we hypothesized that rs2236625 of ADORA2A might be related to the influence of smoking on coronary blood flow. The hypothesis should be validation by further studies with larger patient samples.

Exploration of the HapMap database revealed that the minor allele frequencies of rs7208480 and rs5751879 in the Chinese Han population were both <0.05. The experimental results of this study show that subjects with the rs7208480TT genotype—the distribution of which in the control group deviated from the HWE—constituted only 0.023 in the entire study population. This may be due to the characteristics of genetic drift, since the subjects were from the same geographical area.

This study has produced interesting outcomes and the first evidence of a relationship between *ADORA2A* polymorphism and the severity of CHF in a Chinese Han population; however, several limitations of the study need to be considered. First, the sample was relatively small, and so the reported results need to be validated in a larger sample; Second, the study subjects were drawn from a northern Chinese population, and similar studies in other populations were needed to confirm our findings; Third, we selected one SNP (rs7208480) that was found to be associated with PH by a literature search, without studying all the SNPs of *ADORA2B* [35]; Finally, the functional relevance of the identified polymorphisms in CHF remains to be determined.

## 4. Experimental Section

### 4.1. Patients and Controls

Chinese CHF patients (age > 18 years) were recruited in 2013 and 2014 from the First Affiliated Hospital, College of Medicine, Xi’an Jiaotong University. Patients were classified as having heart failure by experienced cardiologists using the Guidelines for Diagnosis and Treatment of Heart Failure in China (2013) and the cause of the disease was determined according to clinical assessment and echocardiography. The following patient exclusion criteria were applied: age ≤ 18 years, presence of severe hepatic or renal insufficiency, tumors or malignant disease, acute attack of CHF, severe acute infection, metabolic disorders, and acute myocardial infarction without revascularization within the previous 2 weeks. The sample collection was completed according to strict criteria by two visiting staff, and was reviewed by the Chief Physician of Cardiology. Eventually, 300 patients were enrolled.

The healthy subjects, from the Medical Examination Center of the same hospital (age > 18 years) and without known personal or family history of cardiovascular disease, were invited to participate in this study as controls. To identify symptom-free subjects and exclude suspected cardiovascular disease subjects, a detailed interview was conducted in the framework of a physical examination by expert physicians. Four hundred controls were finally included, comparable for sex and ethnicity.

To obtain data on demographic and clinical characteristics, such as gender, age, and traditional cardiovascular risk factors, interviewer-administered health-risk questionnaires were performed. Hypertensive patients were defined according to the Guidelines for Prevention and Treatment of Hypertension in China (2013). Someone taking antihypertensive drugs was also considered to have hypertension. In addition, dyslipidemia and diabetes were defined according to the Guidelines on Prevention and Treatment of Blood Lipid Abnormality in Chinese Adults (2007) and the Guidelines for Prevention and Treatment of Diabetes in China (2013), respectively. Information regarding smoking status was self-reported. Individuals were designated as “smokers” if they reported that they had smoked an average of at least five cigarettes daily for at least the past 12 months [45]. 

The present study was performed with strict protocol under the Ethics Committee of the First Affiliated Hospital, College of Medicine, Xi’an Jiaotong University. A statement of informed consent was obtained from all participants after the procedure had been fully explained to them.

### 4.2. Genotyping Assay

The blood samples were collected into tubes containing ethylenediaminetetraacetic acid. After centrifugation, the samples were stored at −80 °C until analysis. The standard phenol-chloroform extraction method was used to extract genomic DNA from the whole blood. The concentration of DNA was measured by spectrometry (DU530 UV/VIS spectrophotometer, Beckman Instruments, Fullerton, CA, USA). Three tag-SNPs (*i.e.*, rs2236625, rs2236624, and rs4822489), which captured most of the known common variations of ADORA2A according to the Chinese Han population data from HapMap (available online: http://www.hapmap.org), and one SNP (rs5751876) of ADORA2A yielded by a literature search were selected for the present study. In addition, we learned that rs7208480 of *ADORA2B* was associated with PH, which was common in CHF patients, so this SNP was also studied in this study. Sequenom MassARRAY Assay Design 3.0 software (Sequenom Inc., San Diego, CA, USA) was used to design a Multiplexed SNP MassEXTEND assay [46,47,48]. SNP genotyping was performed using the Sequenom MassARRAY RS1000 system according to the standard protocol recommended by the manufacturer [48]. The corresponding primers used for each SNP in the present study are listed in Table 8. Sequenom Typer 4.0 software (Sequenom Inc., San Diego, CA, USA) was used to perform data management and analyses [46,47,48,49]. The association between haplotypes and CHF risk was analyzed using the Haploview soft version 4.2 (Mark Daly’s laboratory, Massachusetts Institute of Technology/Harvard Broad Institute, Cambridge, MA, USA).

**Table 8:** Primers used for this study.

SNP_ID	Allele	1st-PCRP	2nd-PCRP	UEP-SEQ
rs2236625	C/T	ACGTTGGATGTGGTTGGAGGAAATTAACGC	ACGTTGGATGATCTTGGTTAAGGCTACGAC	CTTTAGAGAAAAATCATGTTTTCC
rs2236624	T/C	ACGTTGGATGTCTGGAGGGTGGCTTTACTG	ACGTTGGATGGACAGGGTATGGAGTACAAG	ACAAGAGGCCTAGATCC
rs4822489	T/G	ACGTTGGATGCAGTATGGAAATCCCTGGTC	ACGTTGGATGACACGGGACTTTCTTTGCAG	GAATACTCCCCTTGTGGGTTCCC
rs5751876	T/C	ACGTTGGATGTTGGGCACTCCCTCCACTCA	ACGTTGGATGAGGAGTGTGGGCCAACGGCA	GCGGAGGCCCAATGGCTA
rs7208480	C/T	ACGTTGGATGGCAATGCAGAAGCACATTCG	ACGTTGGATGGTCAGTACTACTGTTAGTGG	GTGGGAGTAGCAATCTTGAA

### 4.3. Statistical Analysis

SPSS 18.0 for Windows (PASW Statistics, SPSS, Chicago, IL, USA) was used for the statistical analyses. Deviation of genotype distributions from Hardy-Weinberg equilibrium (HWE) was determined using the χ^2^-test, which was also used to assess differences in genotype and allele frequencies between study groups. Logistic regression analyses with dominant, recessive, and additive genetic models were used to assess the associations between *ADORA2A* and *ADORA2B* SNPs and CHF.

The association between haplotypes and CHF risk was analyzed using the Haploview soft version 4.2 (Mark Daly’s laboratory, Massachusetts Institute of Technology/Harvard Broad Institute, Cambridge, MA, USA) and haplotype Stats software obtained from Catalan Institute of Oncology (Barcelona, Spain, available online: http://bioinfo.iconcolgia.net).

Differences in parameters related to the severity of CHF, such as ejection fraction (EF), left-ventricular end-diastolic diameter (LVEDD), and left-ventricular end-systolic diameter (LVESD), were evaluated among patients, stratified according to the three different genotypes by analysis of variance, and significant differences among pairs of means were tested using Dunnett’s test. Differences in genotype distribution and allele frequency for the significant SNPs according to functional New York Heart Association (NYHA) class were also analyzed by χ^2^-test. Variables (traditional cardiovascular risk factors) with a retention *p* value of <0.10 in the univariate analysis were entered into a multivariable ordinal regression model to evaluate the association between SNPs and NYHA class. The cutoff for statistical significance was set at *p* < 0.05 (two-sided).

## 5. Conclusions

In conclusion, the findings of this preliminary study have revealed an association between the rs4822489 SNP in *ADORA2A* and the severity of CHF, thus providing novel insight into the contribution of adenosine to heart function. Further functional studies and well-characterized, larger molecular epidemiological studies involving diverse ethnic populations of different countries are necessary to confirm these findings.
